# Conoid tubercle angle: attention should be paid to supraclavicular plate fixation

**DOI:** 10.1186/s13018-022-03003-w

**Published:** 2022-02-19

**Authors:** Bin Zhao, Wenqian Zhao, Isaac Assan, Rongxiu Bi

**Affiliations:** 1 Postdoctoral Research Station, Shandong University of Traditional Chinese Medicine, 4655#, Daxue Road, Changqing District, Jinan, 250355 Shandong Province China; 2 Department of Orthopedics, Shouguang Hospital of Traditional Chinese Medicine, 3353#, Shengcheng Street, Shouguang City, 262700 Shandong Province China; 3 Weifang Key Laboratory for the Prevention and Treatment of Geriatric Diseases, 3353#, Shengcheng Street, Shouguang City, 262700 Shandong Province China; 4 Department of Geriatric Medicine, Qilu Hospital, Cheeloo College of Medicine, Shandong University, 107#, Wenhuaxi Road, Jinan, 250012 Shandong Province China; 5 Department of Traditional Chinese Medicine, The People’s Hospital of Shouguang City, 1233#, Jiankang Street, Shouguang City, 262700 Shandong Province China; 6 College of Medicine and Veterinary Medicine, Deanery of Molecular, Genetic and Population Health Sciences, The University of Edinburgh, Old College South Bridge, Edinburgh, EH8 9YL UK; 7 Department of Orthopedics, Affiliated Hospital of Shandong University of Traditional Chinese Medicine, 16369#, Jingshi Road, Jinan, 250014 Shandong Province China

**Keywords:** Conoid tubercle angle, Pre-contoured anatomic plate, Straight plate

## Abstract

**Background:**

The surgical protocol of ORIF for the treatment of mid-shaft clavicle fractures is common. However, poor plate fit or overhang usually occurs when the straight plate is selected for superior fixation. This is because the upper edge of the clavicle is not flat but has an angulation near the conoid tubercle. We termed that angulation, conoid tubercle angle (CTA). Supposed the straight plate is forcibly attached to the surface of the clavicle, it will potentially cause misalignment of the fracture end and with that comes a change of CTA. In this case, choosing the contoured plate, such as a commercial pre-contoured anatomic plate or manual-contoured plate, for superior fixation seems to meet the requirements for both plate fit and fracture alignment. Hence, we retrospectively compared the radiological parameters, including the plate overhang, and the alignment of the fractures reflected by the CTA, between the contoured plate (CP) and straight plate (SP) groups, to draw attention to the CTA and its effects to supraclavicular plate fixation.

**Methods:**

From March 2018 to April 2021, 217 patients with clavicle fractures that met the inclusion criteria but not the exclusion criteria were included in our study. 112 patients were enrolled into the straight plate group (SP) and 105 patients into the contoured plate group (CP). Besides that, 154 healthy adults were recruited into the health group (HA).

**Results:**

Patients were followed up for 6 to 40 months postoperative. A normal CTA (164.54 ± 4.78°) was obtained from the HA group. There were 50 cases with plate overhang in the SP group, which presented a statistical difference in comparison with the CP group. The value of CTA (169.65 ± 5.84°) in the SP group also indicated a statistical difference in comparison with the normal CTA. Subgroup analysis showed that the CTA (165.88 ± 5.42°) in the overhang subgroup (O) had no statistical difference in comparison with the normal CTA, but the CTA (172.68 ± 4.18°) in the non-overhang subgroup (N-O) had. 3 cases experienced non-traumatic re-fracture (within 3 months after the removal of the fixation) in the O subgroup; 10 cases experienced a poor reduction in the N-O subgroup. In the CP group, the CTA was 166.79 ± 5.68°, which indicated a statistical difference with the SP group. Subgroup analysis was performed, including the manual-contoured plate subgroup (M-C) and commercial pre-contoured anatomic plate subgroup (P-C). The value of CTA (M-C, 166.97 ± 6.33°; P-C, 166.44 ± 6.33°) manifested a statistical difference in comparison with the N-O subgroup. 2 and 8 cases, respectively, had screw loosening and poor reduction in the M-C subgroup. No postoperative complication occurred in the P-C subgroup.

**Conclusion:**

CTA is a useful reference in the evaluation of the reduction obtained on radiographic examination, and a reference guiding the plate contouring. The commercial pre-contoured anatomic plate provides a normal CTA and well fits the biomechanical characteristics of the clavicle, which can be recommended for superior fixation.

## Introduction

Open reduction and internal fixation (ORIF) is a common surgical protocol for the treatment of displaced mid-shaft fractures of the clavicle [1, 2], of which the superior plate is widely used. The clavicle has two inverse curves, and most superior plates are made into an S-shape form from its top view to fix the clavicular curves accordingly [3]. However, the upper margin of the clavicle is not flat, and there is an angulation near the conoid tubercle that can be easily identified in the anteroposterior (AP) view of the clavicle. This was termed Conoid Tubercle Angle (CTA) in this study. To the author’s knowledge, this is the first time to name this angulation. At present, most superior plates are straight and flat from the lateral view. When this kind of plate is fixed across the conoid tubercle, plate overhang is inevitable. Plate overhang causes soft tissue irritation and affects the appearance of the clavicle postoperative. Besides that, it also decreases the strength of the fixation, which can even bring about a failure of the internal fixation. Supposedly, the straight plate is forcibly attached to the surface of the clavicle, misalignment of the fractured ends inevitably occurs, and with that comes a change of CTA. Therefore, the contradiction of plate overhang and fracture alignment seems irreconcilable with a straight plate. Our study aimed to investigate whether this irreconcilable issue can be well solved by the contoured plate, such as commercial pre-contoured anatomic plate and manual-contoured plate. We retrospectively compared the radiological parameters, especially the plate overhang, and the alignment of the fractures that can be reflected by the CTA, between the straight plate (SP, including S-shaped and reconstruction plate) and contoured plate (CP) in the treatment of the mid-shaft fractures, to draw attention to the CTA and its effects on supraclavicular plate fixation.

## Materials and methods

### Patients and healthy adults

From March 2018 to April 2021, 217 patients who met the inclusion criteria but not the exclusion criteria were included in this retrospective cohort study. All were patients with clavicle fractures from the department of orthopedics of Shouguang Hospital of Traditional Chinese Medicine. Besides that, 154 healthy adults were recruited.

### Inclusion criteria

Patients with mid-shaft fracture of the clavicle, who received ORIF with superior plates (Pre-contoured Anatomic Clavicle Plates, Pure Titanium, Suzhou Kangli Orthopaedics instrument CO. Ltd, Suzhou, China; S-shaped and Reconstruction Straight Plates, Pure Titanium, WEGO instrument CO. Ltd, Weihai, China) were enrolled. Patients having associated bilateral clavicle fractures, or other fractures were also included.

### Exclusion criteria

Patients with the plate that did not span the conoid tubercle or less than 5 holes were excluded. Patients with distal clavicle plate or subacromial hook plate were also excluded.

### Demographic and group information

The healthy adults' group (HA) involved 154 healthy adults, 71 females and 83 males. The average age was 45.4 ± 15.5 years (range, 18 to 76 years); The SP group involved 112 patients, 55 females and 57 males. 69 cases with left clavicle fracture and 43 cases with right clavicle fractures. The average age was 49.0 ± 15.3 years (range, 16 to 74 years). 69 cases used the S-shaped straight plate and 43 cases used the reconstruction straight plate; The CP group involved 105 patients, 33 females and 72 males. 50 cases had left clavicle fracture and 46 cases with a right clavicle fracture, including one case with bilateral fractures. The average age was 48.8 ± 15.2 years (range, 17 to 80 years). 33 cases used the manual-contoured S-shaped plate, and 36 cases used the manual-contoured reconstruction plate, while 37 cases used the commercial pre-contouring anatomic plate. All the healthy adults and the postoperative patients received the anteroposterior (AP) view of the clavicle X-ray.

### Follow up and measurement

**Fig. 1:**
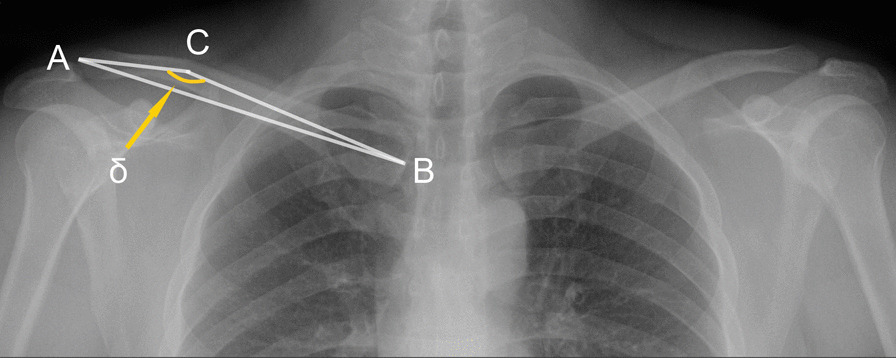
An AP view imaging from a 27-year-old healthy adult. The angle formed by the AC and BC line was the CTA (*δ* = 164°)

**Fig. 2:**
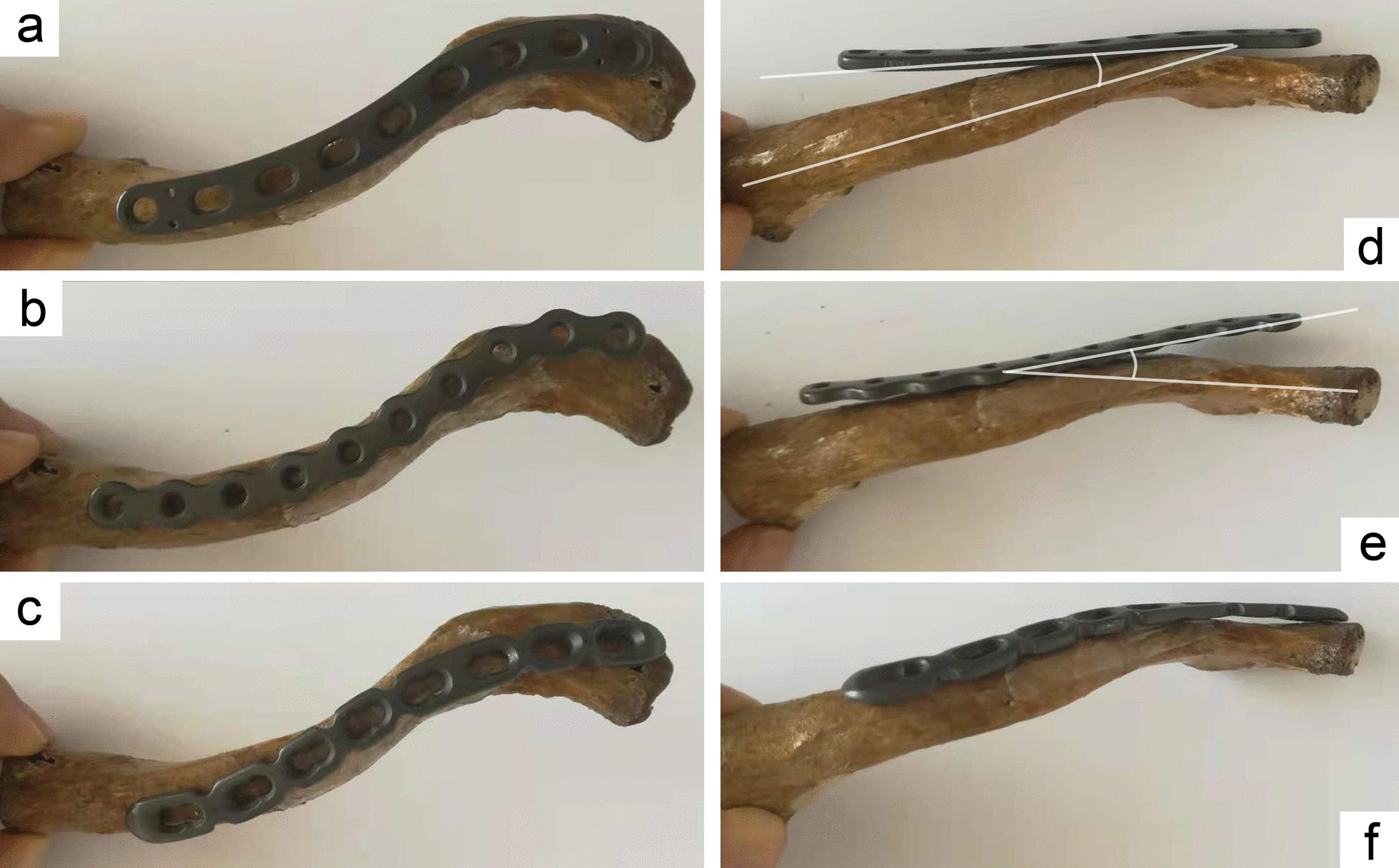
The S-shaped (**a**), reconstruction (**b**), and commercial pre-contoured anatomic plate (**c**) were placed superiorly on the clavicle model. The straight S-shaped and reconstruction plates have plate overhang (**d**, **e**), while the pre-contoured anatomic plate presented a perfect fit to the surface of the clavicle (**f**)

**Fig. 3:**
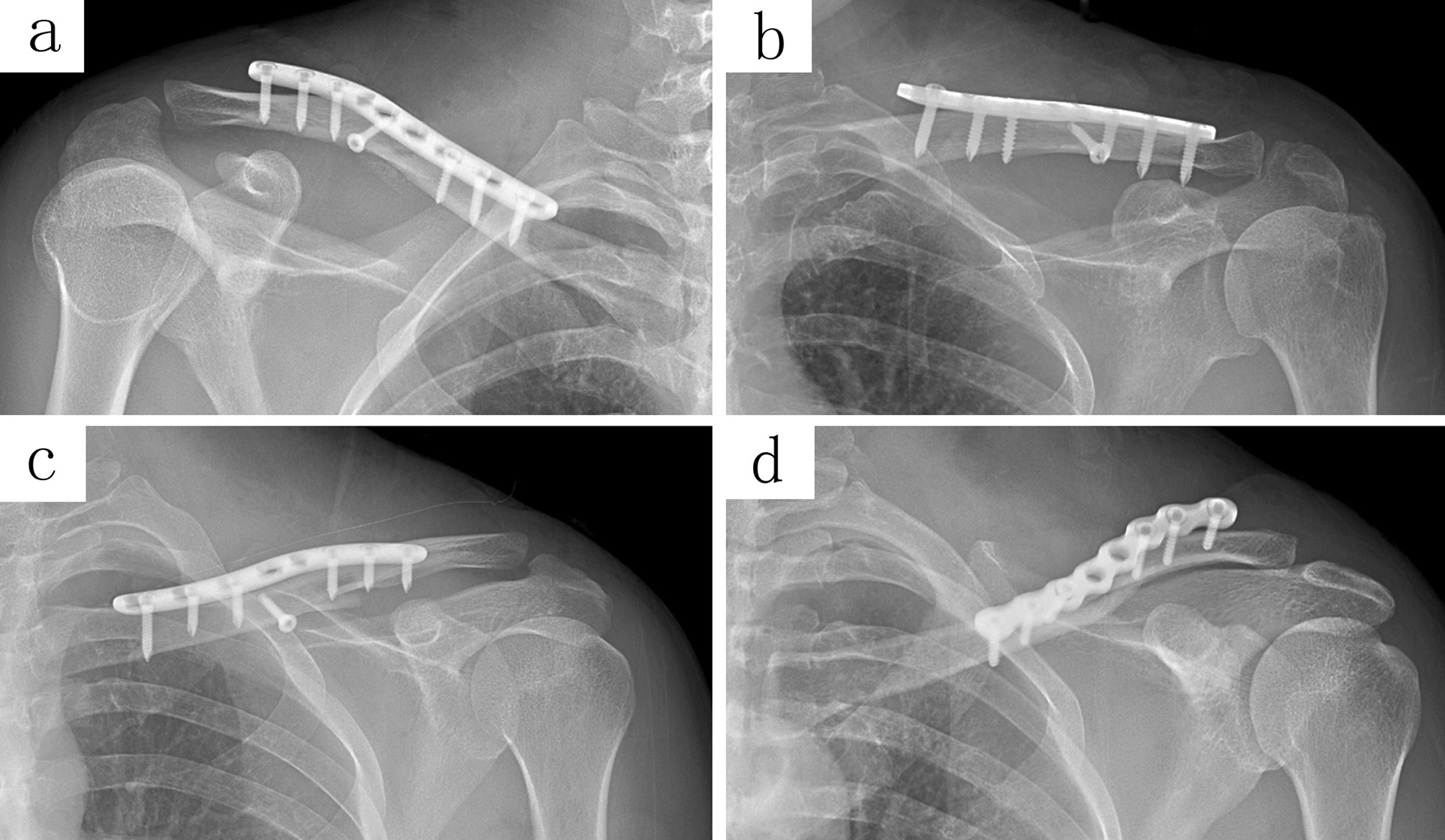
The X-ray imaging manifests the plate overhang (**a**, **b**) or poor reduction (**c**, **d**) of fracture with the straight plate, either in the S-shaped or reconstruction plate

Patients were followed up for 6 to 40 months after ORIF. The radiological parameters were measured with the PACS (Picture Archiving and Communication Systems, version 2.5, Founder Group, Beijing, China). In the AP view of the clavicle, the AB line is the line between the midpoint of the distal and proximal ends; The AC line (distal axis of the clavicle) starts from the midpoint of the distal end and extends along the axis of the distal clavicle cavity; The BC line (proximal axis of the clavicle) starts from the midpoint of the proximal end and extends along the axis of the proximal clavicle cavity. The angle formed by the AC and BC line was the CTA (δ). The bilateral CTA (δ) in healthy adults (Fig. 1) and the CTA of affected sides in the postoperative patient were obtained. Besides that, cases of plate overhang, misalignment of the fracture end, and fixation failure were also measured and recorded. One or more holes overhung from the bone surface (either the acromial or sternal end of the plate), or the angle between the overhanging plate and the axis of the clavicle cavity more than 10°, were considered as a plate overhang (Fig. 2d, e). Cortical misalignment or discontinuity of fracture end more than 2 mm was judged as poor reduction, while the screw loosening, plate breakage, or non-traumatic re-fracture (within 3 months after the removal of the fixation) was regarded as fixation failure (Fig. 3c, d).

### Statistical analysis

Where applicable, data were presented as frequency count or means ± standard deviation. A comparison of data between groups was performed using a one-way analysis of variance (ANOVA), and Least-Significant Difference (LSD) was used for multiple comparisons. The chi-square test was used for the comparison of measurement data. All statistical analyses were performed using the Statistic Package for Social Science (SPSS 19.0). Probability values < 0.05 were considered to be statistically significant. The sample size of the healthy adults was estimated based on the sample size of the infinite population sample.

## Results

The normal CTA (164.54 ± 4.78°, mean value of bilateral; right: 164.65 ± 5.05°, left: 164.42 ± 5.13°) was obtained from the HA group. 50 cases (45.13%) had plate overhang in the SP group, which presented a statistical difference in comparison with the CP group (no case had plate overhang). In the SP group, the CTA was 169.65 ± 5.84°, which indicated a statistical difference in comparison with the normal CTA. Subgroup analysis was performed, including the overhang plate subgroup (O, 50 cases) and non-overhang subgroup (N-O, 62 cases). In the O subgroup, the value of CTA (165.88 ± 5.42°) manifested no statistical difference in comparison with the normal CTA. Besides that, 3 cases experienced non-traumatic re-fracture; In the N-O group, the value of CTA (172.68 ± 4.18°) manifested a statistical difference in comparison with the normal CTA. In addition, 10 cases experienced poor reduction and malunion. In the CP group, no patient experienced plate overhang, and the CTA was 166.79 ± 5.68°, which indicated a statistical difference in comparison with the SP group and normal CTA. Subgroup analysis was performed, including the manual-contouring plate subgroup (M-C, 70 cases) and commercial pre-contouring anatomic plate group (P-C, 36 cases). The value of CTA (M-C: 166.97 ± 6.33°, P-C: 166.44 ± 6.33°) manifested statistical differences in comparison with the normal CTA and N-O subgroup. 2 and 8 cases, respectively, had screw loosening and poor reduction in the M-C subgroup (Table 1).

**Table 1:** The parameters of CTA and complications postoperative

Group	Sex (n)(F/M)	Age(year)	CTA(°)(95%CI)	Type of plate (S/R/P)(n)	Plate overhang(n)	Complications(n)	Subgroup	Sex (n)(F/M)	Age(year)	CTA(°)(95%CI)	Type of plate (S/R/P)(n)	Plate overhang(n)	Complications(n)
HA(154)	71/83	45.40 ± 15.46	164.48 ± 4.85^△^ (163.71 to 165.25)				HA(154)	71/83	45.40 ± 15.46	164.48 ± 4.85^#^ (163.71 to 165.25)			
SP(112)	55/57	48.97 ± 15.27	169.65 ± 5.84*^△^ (168.55 to 170.74)	69/43/0	50^△^	13	O(50)	26/24	49.28 ± 14.80	165.88 ± 5.42(164.34 to 167.42)	25/25/0	50^#^	3
							N-O(62)	28/34	48.73 ± 15.76	172.68 ± 4.18*^#^ (171.62 to 173.74)	44/18/0	0	10
PP(105)	33/72	48.77 ± 15.17	166.79 ± 5.68* (165.69 to 167.28)	33/36/37	0	10	M-C(70)	20/49	49.88 ± 14.97	166.97 ± 6.33*(165.45 to 168.49)	33/36/0	0	10
							P-C(36)	13/23	46.64 ± 16.30	166.44 ± 4.21*(165.01 to 167.86)	0/0/37	0	0

Data were presented as frequency count or means ± standard deviation. A comparison of data between groups including subgroups was performed using a one-way analysis of variance (ANOVA), *P < 0.05 vs. HA, and Least-Significant Difference (LSD) was used for multiple comparisons, ^△^*P* < 0.05 vs. PP. The chi-square test was used for the comparison of fixation failure, ^△^*P* < 0.05 vs. PP, ^#^*P* < 0.05 vs. P-C

There were a total of 13 cases that experienced postoperative complications in the SP group, including non-traumatic re-fracture (3 cases) and poor reduction (10 cases); while 10 cases experienced postoperative complications in the CP group, including 2 cases of screw loosening and 8 cases of poor reduction. The postoperative complication in the O subgroup was mainly reflective in the non-traumatic re-fractures. In the N-O subgroup, it was a poor reduction, while in the M-C subgroup it was screw loosening and poor reduction (Table 2).

**Table 2:** Subgroup analysis for complications postoperative

Subgroup	Fixation failure		Poor reduction
	Screw loosening	Non-traumatic re-fracture	
O(50)	0	3	0
N-O(62)	0	0	10^#^
M-C(70)	2	0	8
P-C(36)	0	0	0

Data were presented as frequency count. The chi-square test was used for the comparison of fixation failure, ^#^*P* < 0.05 vs. P-C

Postoperative complication analysis among different types of plates was made. There were 102 S-shaped straight plates and 79 reconstruction straight plates, being manual-contoured or not, and 37 commercial pre-contoured anatomic plates. The commercial pre-contouring anatomic plates had no postoperative complications. 7 cases had complications with the S-shaped straight plates, and 16 cases had complications with the reconstruction straight plate. The poor reduction rate manifested a statistical difference between the reconstruction straight plate and the commercial pre-contouring anatomic plates (Table 3).

**Table 3:** Postoperative complication analysis with a different type of plate

Type of plate	Fixation failure		Poor reduction
	Screw loosening	Non-traumatic re-fracture	
S(102)	1	0	6
R(79)	1	3	12^#^
P(37)	0	0	0

Data were presented as frequency count. The chi-square test was used for the comparison of fixation failure, ^#^*P* < 0.05 vs. *P*

## Discussion

**Fig. 4:**
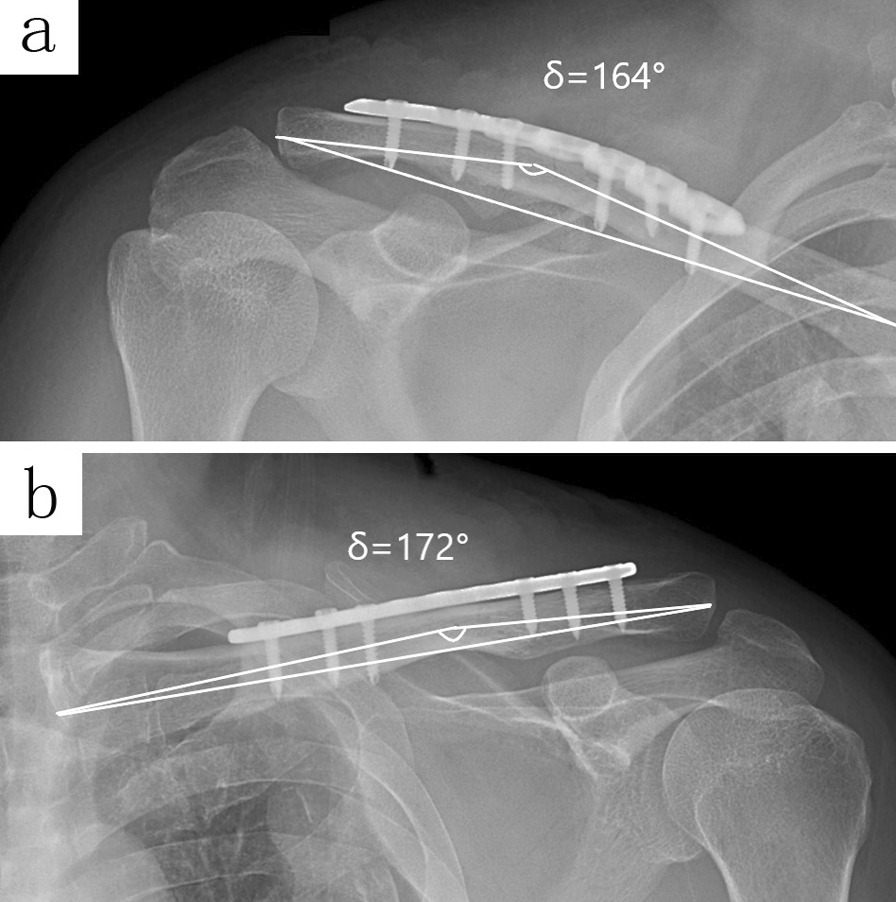
The X-ray imaging shows two healing clavicle fracture cases. The CTA is 164° with a pre-contoured plate (**a**) and 172° with a straight plate (**b**)

A clavicle fracture is a common fracture of the upper limb [4], of which the mid-shaft fracture is common [5]. Since the sternal end is pulled by the sternocleidomastoid muscle, displacement of the fracture end is inevitable. The open fractures, compromised shin, neurovascular complications, mid-shaft fracture displaced or shortened more than 2 cm, comminuted fractures, or additional fractures of the shoulder are indications for surgery [6]. Conservative treatment of the mid-shaft fracture is not within the scope of this study. ORIF is a dominant surgical procedure with a low non-union rate for the treatment of mid-shaft clavicle fracture [2, 7], among which the superior plate is widely used for its biomechanical stability [8]. At present, there is an S-shape straight plate, a reconstruction plate, and the commercial pre-contoured anatomical plate which can be fixed superiorly (Figs. 2a–c and 4).

The clavicle is a long tubular bone. Its acromial end is flat and wide, and the sternal end is gradually enlarged to form a joint with the sternum. The clavicle has two curves forming an S-shape from the top view, with the proximal curve forward and the distal backward, which contributes to the absorbent of stress. The distal one has a greater curvature than the proximal one. Until now, unraveling the anatomy and biomechanical physiology of the clavicle is incomplete. An example is the upper edge of the clavicle which is not completely flat from the AP view but has an angle of 164° near the conoid tubercle. This angulation has been mentioned in previous literature [9], but the relationship between this angulation and the superior plate has not been clarified. Although this study is not perfect, the issue of CTA which reflects the alignment of the fracture and its effects on superior plate fixation still merits discussion. To the author’s knowledge, this is the first primary research referring to the CTA and its influence on the fixation of the superior clavicle plate.

In this study, the value of CTA in the SP group manifested statistical differences when compared with the CP group or normal group. The value of CTA in the N–O subgroup manifested statistical differences when compared with the O subgroup and the normal group. The value of CTA in the O subgroup manifested no statistical differences in comparison with the normal group. In other words, in the SP group, supposed the requirements of the normal CTA are met, plate overhang occurrence is inevitable. This has been confirmed in the O subgroup. Supposed the requirements of plate fit are met, the CTA will be compromised, and as a result, misalignment of the fractured end will occur. In this study, we obtained the normal CTA from healthy adults, rather than from the patient's, for the following reasons: The CTA of the affected clavicle could not be obtained before surgery; The CTA in both sides of the clavicle is not identical even in healthy adults. This was confirmed when the bilateral CTA was measured in healthy adults. Thus, we took the mean value of bilateral as the normal CTA to reduce the measurement bias.

**Fig. 5:**
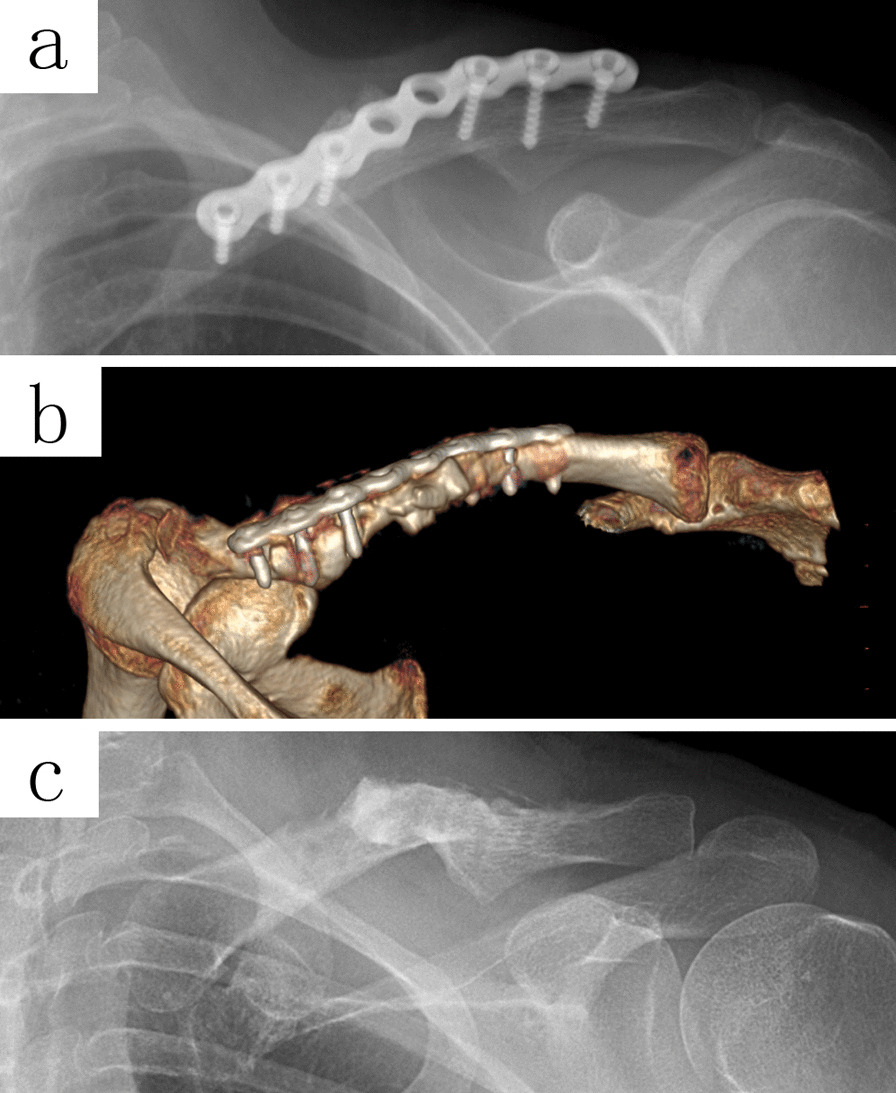
The X-ray imaging shows the plate overhang (**a**), and the CT imaging confirmed the screw deviation from the center of the bone (**b**). Finally, the patient experienced screw loosening and malunion (**c**)

With the existence of the CTA, when the straight plate was fixed across the conoid tubercle, the plate could not achieve an adequate fit, and an overhang occurred (Figs. 2d, e and 3a, b). Subcutaneous prominence caused by plate overhang is a disadvantage of the superior plate. Therefore, many patients require plate removal after the fracture has healed [10]. This study found out that, the superior straight plates, whether S-shaped or reconstruction plates, were prone to overhang from the bone surface than that of pre-contoured plates, and the plate overhang could easily be identified by palpation in emaciated patients. If these kinds of plates are forcibly fit to the surface of the clavicle, the CTA will increase (Fig. 4b), resulting in biomechanical changes of the clavicle, and even causing poor reduction of the fracture ends (Fig. 3c, d). When the plate overhang occurs, the overhang side of the plate needs to be fixed with longer screws. However, it is not so simple to solve the overhang problem with longer screws. Attention should be paid to the following issues: screw deviation from the center of the bone (Fig. 5a, b), vacant hole without proper screw to drill in, screw loosening or screw pullout, stress fractures occurring distal to the plate in an attempt to fit the bone with a regular lag screw.

**Fig. 6:**
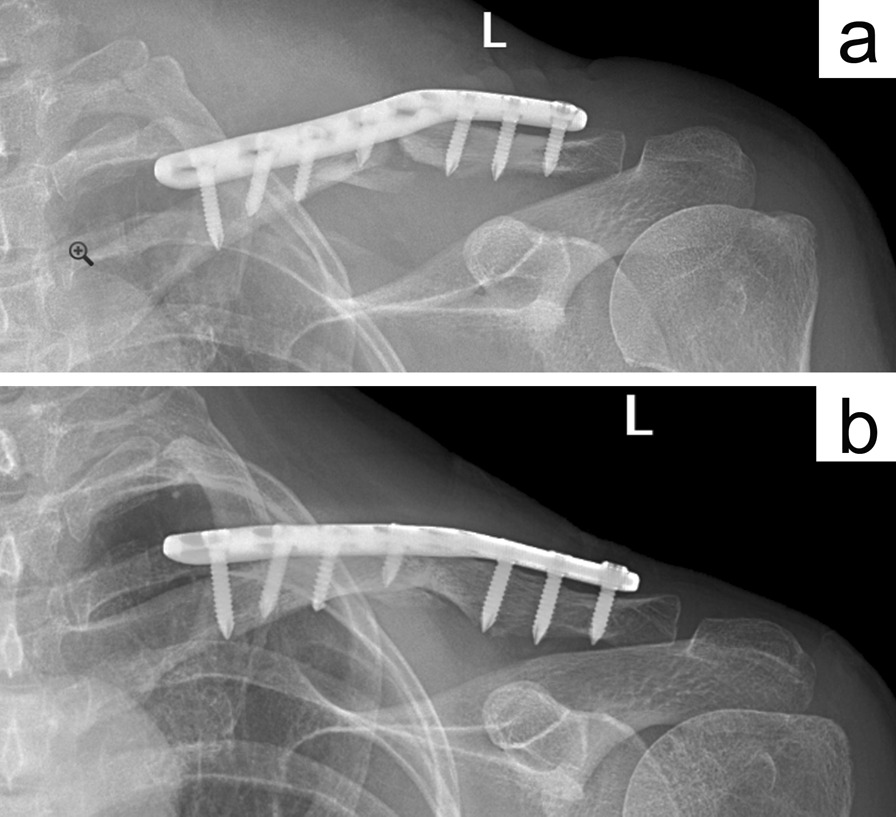
The X-ray imaging showed that a case of clavicle fracture was fixed with an incorrect manual-contoured straight plate, as a result, both the plate overhang and poor reduction occurred (**a**). Finally, the patient experienced plate overhang and malunion at the last follow-up (**b**)

A finite study [11] reported that the averaged plate-like area of the clavicle is like a twisted strip from upwards of the acromial end to the anterior of the sternal end and is also the major stress concentration area of the clavicle. Accordingly, the superior plate system requires three-dimensional pre-contoured to achieve a satisfying fit, in this case, the commercial pro-contoured commercial plate (Fig. 4a) not only provides a normal CTA but also a twist-shape to coincide with the biomechanical characteristics of the clavicle, thereby, reducing the plate overhang and potential soft tissue irritation. Unlike the commercial pre-contoured plate, straight plates or reconstruction plates are difficult to obtain a good fit by manual-contoured; on the contrary, a manual-contoured deviation damages the alignment of the fracture, even causing a higher poor reduction rate in this study (Fig. 6) (Table 3). In this case, commercial pre-contoured anatomic plates possess inherent advantages which can be recommended for superior clavicle fixation.

## Conclusion

CTA is a useful reference in the evaluation of the reduction obtained on radiographic examination, and a reference guiding the plate contouring. The commercial pre-contoured anatomic plate provides a normal CTA and well fits the biomechanical characteristics of the clavicle, which can be recommended for superior fixation.

## Data Availability

The datasets used and/or analyzed during the current study are available from the corresponding author on reasonable request.

## Abbreviations

CTA: Conoid tubercle angle
CP: Contoured plate
SP: Straight plate
HA: Healthy adults
O: Overhang subgroup
N-O: Non-overhang subgroup
ORIF: Open reduction and internal fixation
AP: Anteroposterior
ANOVA: One-way analysis of variance
LSD: Least-significant difference
SPSS: Statistic Package for Social Science
S: S-shaped plate
R: Reconstruction plate
C: Commercial pre-contoured anatomic plate
M-C: Manual-contoured plate subgroup
P-C: Commercial pre-contoured anatomic plate subgroup
